# Development and Validation of a Combined RT-LAMP Assay for the Rapid and Sensitive Detection of Dengue Virus in Clinical Samples from Colombia

**DOI:** 10.3390/diagnostics15050570

**Published:** 2025-02-27

**Authors:** Leidy Hurtado-Gómez, Katherine Escorcia-Lindo, Juan Sebastian Rosero, Nataly Solano Llanos, Camilo Barrios Sánchez, Anderson Díaz Pérez, Yirys Díaz-Olmos, Jennifer García, Yesit Bello-Lemus, Leonardo C. Pacheco-Londoño, Antonio J. Acosta Hoyos, Lisandro A. Pacheco-Lugo

**Affiliations:** 1 Centro de Investigaciones en Ciencias de la Vida, Facultad de Ciencias Básicas y Biomédicas, Universidad Simón Bolívar, Barranquilla 080001, Colombia; leidy.hurtado@unisimon.edu.co (L.H.-G.); katherineescorcia52@gmail.com (K.E.-L.); jrosero@mail.uniatlantico.edu.co (J.S.R.); nataly.solano@unisimon.edu.co (N.S.L.); camilo.barrioss@unisimon.edu.co (C.B.S.); anderson.diaz@unisimon.edu.co (A.D.P.); yesit.bello@unisimon.edu.co (Y.B.-L.); leonardo.pacheco@unisimon.edu.co (L.C.P.-L.); antonio.acosta@unisimon.edu.co (A.J.A.H.); 2 Programa de Medicina, Grupo GINUMED, Facultad Ciencias de la Salud, Corporación Universitaria Rafael Núñez, Cartagena de Indias 130001, Colombia; 3 División Ciencias de la Salud, Universidad del Norte, Barranquilla 080001, Colombia; olmosy@uninorte.edu.co; 4 Clínica General del Norte, Barranquilla 080001, Colombia; jennifer.garcia@zentria.com.co

**Keywords:** TURN-RT-LAMP, dengue virus (DENV), colorimetric test, point of care

## Abstract

**Background**: Dengue virus (DENV) infection is a significant public health concern in several tropical and subtropical regions, where early and rapid detection is crucial for effective patient management and controlling the spread of the disease. Particularly in resource-limited, rural healthcare settings where dengue is endemic, there exists a need for diagnostic methods that are both easy to perform and highly sensitive. **Objective**: This study focuses on the development and validation of a single-tube reverse transcription loop-mediated isothermal amplification termed TURN-RT-loop-mediated isothermal amplification (LAMP) for the detection of DENV. **Methodology**: The TURN-RT-LAMP assay designed in this study combines two sets of primers targeting the 5′- and 3′-UTR of DENV, with the aim to increase the sensitivity of detection. **Results**: Clinical validation of the TURN-RT-LAMP assay using samples collected from febrile individuals with a serological or antigenic diagnosis revealed a sensitivity of >96%. The performance of this assay was statistically compared with that of the standard diagnostic method, quantitative reverse transcription-polymerase chain reaction. **Conclusions**: The results support the potential of RT-LAMP as a rapid, sensitive, and specific tool for the diagnosis and surveillance of dengue, particularly suitable for field use in low-resource settings.

## 1. Introduction

Dengue virus (DENV) infection has shown a concerning increase in the number of cases over recent decades, posing a major threat to global public health. DENV infection affects people of all ages, including infants, children, and adults, placing a heavy burden on healthcare systems. The World Health Organization reports that there are approximately 390 million dengue infections each year, with 96 million exhibiting clinical symptoms. Although dengue is endemic in more than 100 countries, primarily in the Americas, Southeast Asia, and the Western Pacific, its presence has also spread to unexpected regions in Europe [1,2].

DENV, which belongs to the Flaviviridae family, is considered the most prevalent arbovirus worldwide. Its impact on public health is unquestionable, and there are still numerous challenges in controlling it, particularly in tropical and subtropical regions [3].

DENV has four distinct serotypes, DENV-1, DENV-2, DENV-3, and DENV-4, which are antigenically related. Like other flaviviruses, these serotypes possess a single-stranded RNA genome encased within an icosahedral structure and surrounded by a lipid envelope [4]. DENV is transmitted to humans by the bite of an infected female mosquito of the *Aedes* genus [5]; however, the widespread distribution and high adaptive capacity of *Aedes albopictus* suggest that this vector could also become an active transmitter of dengue in endemic regions [6]. During the usual 5-day period of viremia, the mosquitoes become infected when they feed on viremic humans. The virus is transferred from the intestinal tract of the mosquito to the salivary glands after an extrinsic incubation period, a process that takes approximately 10 days and is most rapid when the ambient temperature is high [7].

The lack of specific treatments for dengue and the need for early detection emphasize the importance of developing and improving molecular diagnostic tools. Traditional methods, such as indirect immunofluorescence and serological tests, have significant limitations, including the need for a second serum sample to confirm results and the persistence of detectable antibodies for a lifetime. These issues are further complicated by cross-reactivity in diagnostic tests, as reported by the Centers for Disease Control and Prevention [1]. In response to these limitations, molecular techniques such as RT-PCR and RT-qPCR have emerged as promising alternatives for diagnosing dengue, providing greater sensitivity and speed. Nevertheless, the complexity and high costs associated with these methods present challenges for large-scale accessibility and application. In recent years, there has been significant progress in the development and optimization of isothermal-based methodologies for the molecular diagnosis of DENV and other arboviruses, such as Zika and chikungunya. Among these emerging methodologies, the loop-mediated isothermal amplification (LAMP) test is remarkable for its speed, sensitivity, specificity, low cost, and suitability for use in primary healthcare centers with limited infrastructure [8]. LAMP is an isothermal amplification test, proposed by Notomi, which uses three pairs of primers and the enzyme Bst (*Bacillus stearothermophilus*) working at temperatures between 60 °C and 65 °C. The LAMP method is based on two or three primer pairs forming a dumbbell-shaped structure during synthesis under the action of Bst polymerase, with the structure obtained to serve as a template for further amplification. The resulting LAMP products are concatemers containing multiple copies of that stem structure [8,9]. RT-LAMP is a variant of LAMP in which reverse transcriptase is incorporated to amplify RNA targets. In this method, reverse transcriptase is added to the reaction to convert RNA into DNA, allowing for subsequent amplification. Because it involves reverse transcription, the technique is referred to as RT-LAMP [10,11].

In this study, we investigated the usefulness of a two UTR recognition LAMP test for nucleic acid amplification of DENV in Colombia and demonstrated that this approach, referred to here as TURN-RT-LAMP, is highly sensitive, making it a better alternative for implementation as a point-of-care test in areas with high dengue endemicity in Colombia. This study is not only aligned with the objectives of the Comprehensive Health Care Model (MIAS), which places people and their well-being at the center of care, but also seeks to optimize and standardize the diagnosis of dengue from educational, social, and political perspectives. The standardization of these techniques not only improves the training of health professionals but also exerts a positive impact on patient care and facilitates decision-making based on accurate epidemiological data.

## 2. Materials and Methods

### 2.1. Type of Study

This was a cross-sectional study conducted to evaluate a dual UTR RT-LAMP (TURN-RT-LAMP) assay for the detection of DENV in three phases, viz., (a) standardization, (b) laboratory validation, and (c) field validation. qRT-PCR was used as the reference standard.

### 2.2. Samples

A total of 158 serological samples were collected from suspected DENV cases at Clínica General del Norte in Barranquilla city and sent to the Life Science Research Center (CICV) of the Simón Bolívar University in Barranquilla for further processing and molecular detection of DENV.

### 2.3. RNA Extraction

All the 158 samples were subjected to RNA extraction from 200 μL of serum using the Quick-RNA Viral Kit™ extraction kit (ZYMO Research, Irvine, CA, USA; catalog: R2141-E), which uses silica magnetic beads for total RNA purification, according to the manufacturer’s recommendations. Briefly, 400 μL of viral RNA buffer was added to each 200 μL of sample (2:1) and mixed gently. Then, successive washes were performed to finally elute the RNA in 15 μL of DNase/RNase-free water.

### 2.4. RT-qPCR

The SuperScript III One-Step RT-PCR System with the Platinum Taq DNA Polymerase™ kit (Invitrogen, Carlsbad, CA, USA; catalog: 1257401) was used to perform RT-qPCR. Briefly, 8 μL of 2× reaction buffer containing 0.4 mM of each dNTP, 3.2 mM of MgSO_4_, 0.3 μL of reverse transcriptase (SuperScript III RT/Platinum Taq Mix), 200 nM of each primer directed against different genes of DENV, and 100 nM of each fluorophore-conjugated TaqMan probe were used. The molecular diagnosis protocol of DENV reported by Nunes was used [12] (Table 1). RT-qPCR was conducted using the CFX96™ Bio-Rad kit under the following amplification conditions: 50 °C for 20 min, followed by 95 °C for 3 min, and ending with 45 cycles of 95 °C for 15 s and 58 °C for 30 s.

### 2.5. Turn-RT-Lamp

**Figure 1:**
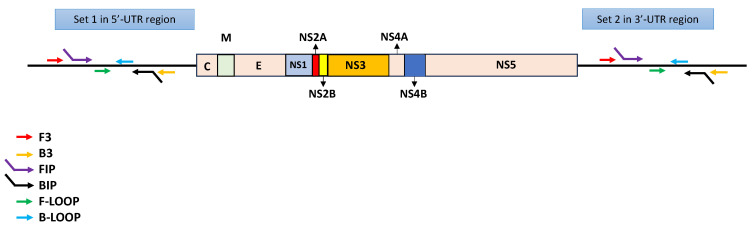
Diagram depicting the TURN-RT-LAMP test. It depicts the 5′- and 3′-UTR recognized by Set 1 and Set 2, respectively.

The TURN-RT-LAMP isothermal amplification assay was conducted using the Warmstart Colorimetric LAMP 2× Master Mix (DNA and RNA) kit obtained from New England Biolabs, Ipswich, MA, USA), according to the manufacturer’s instructions. In this study, the primers reported by Dauner et al. [13] targeting the conserved 5ʹUTR of DENV and the primers reported by Teoh et al. [14] targeting the 3′-UTR of DENV were validated in a single reaction [13,14] (Figure 1). The primer sequences are shown in Table 2.

Before each LAMP reaction, a 10× mixture of single-use primers was prepared, and a short incubation for 5 min at 95 °C was performed to eliminate any formation of secondary structures that could impact the color change of the reaction [10] (Table 2).

**Figure 2:**
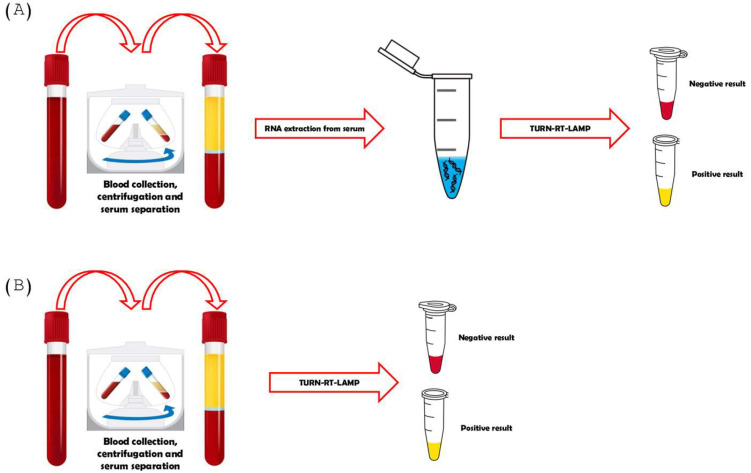
The TURN-RT-LAMP test for DENV diagnosis. RT-LAMP test with (**A**) previous RNA extraction and (**B**) without previous RNA extraction.

The LAMP reaction mixture was prepared using 10 μL of Warmstart colorimetric reagent LAMP 2× Master Mix (DNA and RNA), 5 μL of water, 3 μL of the previously extracted RNA, and 2 μL of the 10× master mix of the respective primer set that had been previously incubated at 95 °C. The 10× primer mixture was always added through the lid of the reaction tube to avoid unspecific amplifications that could start before the optimal amplification temperature was reached. The reaction was started by a rapid spinning of the tubes and an incubation for 30 min at 65 °C in a thermal cycler (SimpliAmp Thermal Cycler™, Thermo Fisher Scientific, Waltham, MA, USA). After incubation, the result was visualized by colorimetry. The color change from pink to yellow was indicative of a positive reaction. Figure 2A depicts pink and yellow samples, wherein pink corresponds to negative samples and yellow corresponds to positive samples for DENV.

### 2.6. Turn-RT-Lamp to Detect DENV in Samples Without Prior Extraction

We applied a simple human blood lysis procedure for the LAMP reaction to simplify the DNA/RNA preparation process. Briefly, 100–200 µL of human blood collected in a heparin tube was transferred to a tube containing 900 µL of blood lysis buffer (0.1% Triton X-100 in DDW), vortexed for 1 min, and then allowed to stand for 30 min. For the LAMP reaction, 10 µL of lysed blood was used, which was performed as usual (Figure 2B).

### 2.7. Data Analysis

Statistical analyses were conducted using the statistical software RStudio version (4.4.2). The specificity of the RT-LAMP test was evaluated using the fraction of RT-qPCR-negative samples that were also negative in the RT-LAMP assays. Sensitivity was expressed as the fraction of samples that were positive for RT-qPCR that were also positive for RT-LAMP. Positive predictive and negative predictive values were calculated as follows:

Positive predictive value (PPV):
  •VPP = A/(A + B)
Negative predictive value (NPV):
  •NPV = D/(C + D)

The kappa index was calculated to estimate the concordance of RT-LAMP results with RT-qPCR results, as shown in Table 3.

$$k=\frac{P0-Pe}{1-Pe}$$

*P0*: Probability of agreement observed
*Pe*: Probability of agreement by chance

## 3. Results

This study aimed to explore the utility of two previously published RT-LAMP protocols for the rapid diagnosis of DENV infection in samples collected from febrile patients with a presumptive serological or antigenic diagnosis of dengue [13,14]. We speculated if combining both protocols using the primer sets reported in each study could improve the sensitivity of the method in samples collected from Colombian patients.

We investigated the performance of the TURN-RT-LAMP protocol in 158 samples previously diagnosed as DENV-positive by the IgM or NS1 antigen test. RNA was extracted from these samples, and they were confirmed as DENV-positive by RT-qPCR using the CDC protocol with primers targeting the E gene [12]. In parallel, we evaluated the samples using the TURN-RT-LAMP method, which amplifies two conserved sequences in the 5ʹ- and 3ʹ-UTR of the DENV genome (Figure 1). We first examined a conventional RT-LAMP protocol using the RNA extracted from the serum samples of febrile patients as the starting material (Figure 2A). Then, we generated a calibration curve to determine the copy number in relation to Ct values, for which we used a DENV-2 control and performed serial dilutions. The formula and curve were prepared according to the methodology described by Hurtado et al. [10], as shown in Table 4.

**Figure 3:**
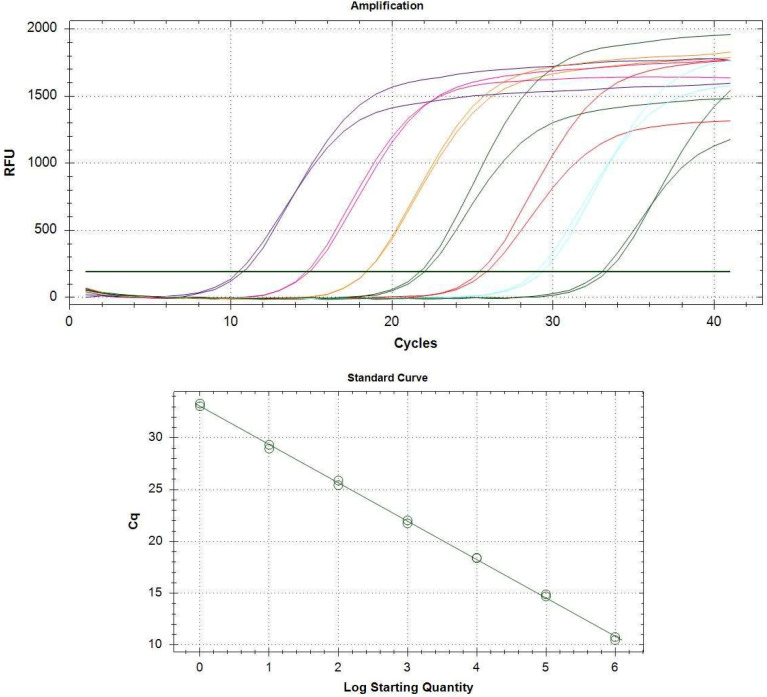
Amplification and standard curve. The amplification efficiency was 86.3%. R^2^ was 0.99. Standard curve and amplification from CFX Maestro Software. The amplification graph displays lines in different colors, with each line representing a specific dilution. For each dilution, two lines of the same color are shown, corresponding to the technical replicates performed for that dilution.

The standard curve was generated through linear regression analysis of the Ct values obtained from real-time PCR for each amplification reaction against the log_10_ RNA copy number of DEN-2 (Figure 3 and Table 4). This enabled us to determine the copy numbers in the samples, as shown in Table S1 (Supplementary Materials).

The standard curve showed an R^2^ of 0.999, an amplification efficiency of 86.3%, and a slope of −3.702. These values indicate a slope close to the ideal and an efficiency within the acceptable range of 90–110%; it is 1.863 of the range duplication each cycle (Figure 3).

In total, 51 samples were RT-qPCR-positive, and 107 were RT-qPCR-negative, whereas 47 samples were TURN-RT-LAMP-positive, and 111 were TURN-RT-LAMP-negative. There were 152 concordant samples and 8 discordant samples.

**Figure 4:**
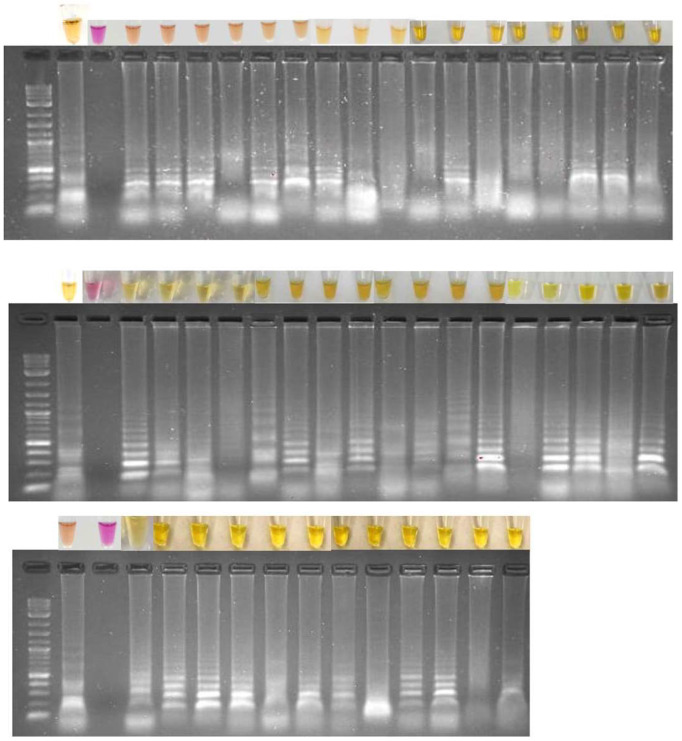
Electrophoretic run of the TURN-RT-LAMP colorimetric reaction for the diagnosis of DENV. The presence of bands of different sizes, characteristic of a LAMP-positive sample, in this case positive for DENV, is observed, whereas the absence of bands indicates a negative sample. The respective colorimetric result of the LAMP reaction is shown at the top.

The results of the colorimetric TURN-RT-LAMP test for DENV are shown in Figure 4. The reaction was performed using the Warmstart colorimetric reagent (NEB), two sets of primers shown in Table 2, and samples with suspected DENV infection. Water was included as a negative control, and a DENV isolate from infected cell cultures was included as a positive control. Figure 4 depicts pink and yellow samples, where pink corresponds to negative samples and yellow corresponds to positive samples for DENV. Electrophoresis was performed to corroborate the results by observing the electrophoretic run. LAMP-positive samples exhibit a banding pattern characteristic of the positive samples, because the LAMP reaction generates fragments of different sizes, whereas the negative samples do not exhibit any banding. Therefore, the colorimetric results correspond with the electrophoretic runs.

The sensitivity and specificity of the TURN-RT-LAMP test were determined in comparison with RT-qPCR. A sensitivity of 0.96 was achieved, indicating that samples positive for RT-qPCR were also positive for TURN-RT-LAMP. A specificity of 0.95 was also obtained, implying that the TURN-RT-LAMP test correctly identified 95% of the RT-qPCR-negative samples. These results are summarized in a contingency table (Table 5).

As shown in Table 5, of the 158 samples tested, 45 were true positives, 105 were true negatives, 6 were false positives, and 2 were false negatives.

The TURN-RT-LAMP technique for DENV diagnosis using the primer sets demonstrated a sensitivity of 0.96, suggesting that this technique was efficient in detecting DENV-positive samples, as shown below:

$$Sensitivity=\frac{45}{47}=0.96$$

The specificity for the TURN-RT-LAMP technique was 0.96, i.e., it was specific for detecting negative samples.

$$Specificity=\frac{105}{111}=0.95$$

In the statistical analyses conducted using RStudio, the PPV of TURN-RT-LAMP was approximately 0.88 (0.76, 0.96), and the NPV was approximately 0.98 (0.93, 1.00). The TURN-RT-LAMP technique applied for detecting DENV shows promising results as it is an easy-to-use diagnostic technique performed in less time and with the characteristics of sensitivity and specificity that are competitive with the gold standard. The concordance was analyzed through the kappa index (κ) for TURN-RT-LAMP, the formula and interpretation of the Cohen kappa coefficient, defined as a function of the proportion of observed agreement and proportion of chance agreement (Table 3). The kappa index was 0.88, indicating that the TURN-RT-LAMP results were almost in perfect agreement with DENV PCR results. The Po was 0.949, and Pe was 0.571. These values demonstrated the possibility to detect DENV using the TURN-RT-LAMP technique, which was almost in perfect agreement with the gold standard test. The TURN-RT-LAMP assay is extremely sensitive and specific for diagnosing DENV. Table 6 summarizes the results of the TURN-RT-LAMP test.

We calculated the detection limit of the TURN-RT-LAMP test for the samples included in this study by considering the lowest copy number present in a sample that tested positive in the technique. The TURN-RT-LAMP test showed a detection limit for samples with a copy number close to five copies and Cq values > 34. This result supports the sensitivity of the technique in the clinically relevant range for DENV detection. In relation to the viral copy number, the sensitivity of the TURN-RT-LAMP test for samples with more than 1.3 × 10^2^ copies was one. For samples between 1.3 × 10^2^ and 5 copies, the sensitivity was 0.95. The TURN-RT-LAMP test was extremely sensitive for detecting DENV in samples with a viral load of less than 100 copies (Table 7). According to the viral copy number, samples with a viral load higher than 1.3 × 10^2^ copies showed concordance in both TURN-RT-LAMP and RT-qPCR tests. In the analysis performed, we found variability in the Cq values, indicating differences in the viral loads of the DENV samples analyzed by TURN-RT-LAMP. Samples discordant between the two methods showed Cq values higher than 30, which corresponds to those samples with a viral load equal to or less than 100 copies. As previously mentioned, TURN-RT-LAMP proved to be sensitive for the detection of samples with viral loads above 100 copies. This suggests that the technique is effective for samples with low Cq values. As we mentioned, TURN-RT-LAMP was sensitive enough to detect samples above 100 copies. Cq and copy number in discordant samples are shown in Table S1.

**Figure 5:**
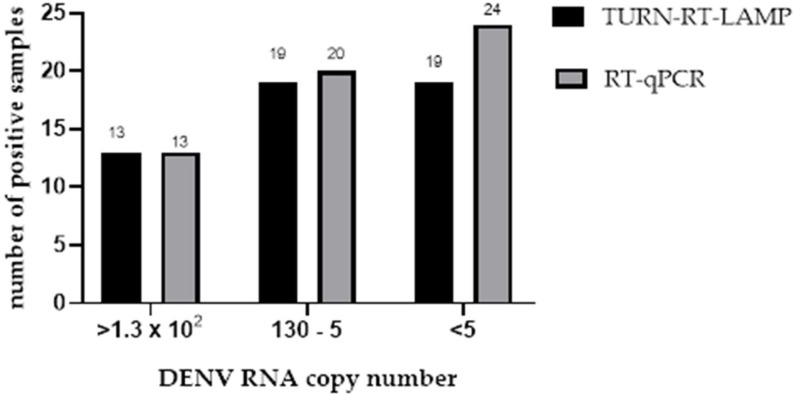
Samples positive for TURN-RT-LAMP and RT-qPCR in relation to DENV RNA copy number.

As shown in Figure 5, thirteen samples tested positive in both assays. For samples with a viral load ranging between 1.3 × 10^2^ and 5 copies, high sensitivity was observed; however, the results were not fully concordant between the two techniques. As the viral load decreased, the sensitivity of the LAMP test also diminished. For samples with viral loads higher than five copies, the results were inconsistent between the two methods, with 24 samples testing positive by RT-qPCR and 19 samples testing positive by LAMP (Figure 5, Table 7).

Given that these isothermal approaches must be practical for use in primary care settings, we investigated the feasibility of applying the TURN-RT-LAMP technique directly to serum samples collected from four febrile patients, bypassing RNA extraction. Briefly, 100 µL of human blood was lysed using a lysis buffer (0.1% Triton X-100 in DDW), vortexed for 1 min, and left to stand for 30 min. The TURN-RT-LAMP assay was performed at 65 °C for 45 min. Four blind samples from the experimental set (DENV-CGN-012, DENV-CGN-014, DENV-CGN-032, and DENV-CGN-033) were analyzed using the TURN-RT-LAMP assay without prior nucleic acid extraction. All four samples tested positive for DENV (Supplementary Figure S1). These results were validated by RT-qPCR, confirming that the samples were DENV-positive. Quantitative analysis determined that the copy numbers in these samples ranged from 1.3 × 10^2^ to 5 copies, with Cq values between 20 and 30. All these samples tested positive with the TURN-RT-LAMP assay.

Heparin is a concomitant inhibitor of DNA polymerases and interferes with nucleic acid amplification; nevertheless, our results demonstrated that heparin did not inhibit the LAMP reaction because we used Triton X-100 to lyse the cells. Using a detergent (e.g., Tween 20 or Triton X-100) or incorporating chelating agents neutralizes the inhibitory activity of heparin. Therefore, we used Bst polymerase, which has a lower sensitivity to inhibitors than the Taq polymerase used in conventional PCR. LAMP offers several advantages over traditional PCR, including reduced sensitivity to common inhibitors like ethanol, isopropanol, EDTA, and sodium acetate [13]. Hence, the TURN-RT-LAMP assay can be successfully used for the direct detection of DENV in heparin-containing samples without the need for extensive nucleic acid extraction [8,15].

## 4. Discussion

Dengue is a serious global public health issue, exacerbated by climatic and migratory phenomena that have expanded the range of the vector *A. aegypti*. The World Health Organization, through the ASSURED criteria, has urged scientists to develop molecular diagnostic methods that can overcome the limitations of PCR and real-time PCR [16]. This call has resulted in the development of various molecular diagnostic methodologies for DENV, with enormous potential for use in low-resource settings. Among these methods, LAMP has gained significant importance due to its simplicity, sensitivity, specificity, low cost, and suitability for use in rural areas with inadequate infrastructure. For DENV, several protocols have been reported to date, each with its own unique features and efficiencies [13,14,17,18,19,20,21,22,23].

Nevertheless, existing protocols are often less reproducible when applied to different geographic regions, with less common serotypes, or even different viral genotypes within each serotype. Therefore, it is necessary to optimize these protocols for each region to establish their utility as a reproducible molecular test on a global scale.

The present study was conducted to validate the efficacy of two previously reported RT-LAMP protocols (in Peru and Malaysia) for dengue diagnosis in an endemic area in Colombia [13,14]. Although both protocols had demonstrated high sensitivity and specificity in studies conducted in Peru and Malaysia, respectively, they did not replicate this performance in our study population. The sensitivity and specificity obtained by applying each protocol separately were unsatisfactory, which led us to investigate the possibility of combining both approaches to improve the diagnostic accuracy. When we combined both protocols into an integrated approach targeting both UTRs, we obtained interesting results. In fact, the concordance between TURN-RT-LAMP and RT-qPCR results was high (152 of 158 samples), with a sensitivity and specificity of 0.96, suggesting that TURN-RT-LAMP has the potential to be used as a diagnostic tool for dengue in Colombia.

In our study, the TURN-RT-LAMP test detected 47 positive cases among 158 suspected DENV cases, while RT-qPCR identified 51 positives, achieving a high accuracy (152/158) between the two methods. The sensitivity differences between RT-qPCR and LAMP were primarily due to viral load variations; as RNA levels decreased, LAMP sensitivity decreased correspondingly (Figure 5, Table 6). The 3′-UTR of DENV is a highly conserved sequence across the four circulating serotypes worldwide, making it a common diagnostic target. However, serotype 4 exhibits some variations in this region, necessitating a specific primer set for accurate detection. A likely reason for the lower sensitivity of the primer sets reported by Dauner and Teoh, compared to our results, is that their studies did not include clinical samples from serotype 4. Their samples consisted of DENV-1, DENV-2, and DENV-3, which were naturally circulating in northern Peru at the time of collection. In contrast, our study included multiple clinical samples from serotype 4.

In the study by Dauner et al., the optimized RT-LAMP assay demonstrated a sensitivity of 86.3% (95% confidence interval [CI], 72.7–94.8%) and a specificity of 93.0% (95% CI, 83.0–98.1%). Teoh et al. reported that RT-LAMP could detect DENV with fewer than 10 viral RNA copies. Their evaluation, conducted using serially diluted viral RNA extracted from culture supernatant, showed positive detection rates of 100% (12/12) for RNA copy numbers of 1000 and 100, 75% (9/12) for 60 copies, and 25% (3/12) for 10 copies. The specificity of the assay was further validated by digesting the amplified DNA fragments from all four DENV serotypes with the restriction enzyme BanII. The digested DNA fragment sizes matched the expected sizes for each serotype: 129 bp and 233 bp for DENV-1, 135 bp and 231 bp for DENV-2, 129 bp and 231 bp for DENV-3, and 141 bp and 231 bp for DENV-4. Nucleotide sequencing of the 231 bp and 233 bp digested fragments confirmed that the RT-LAMP-amplified sequences were specific to DENV. Overall, the RT-LAMP assay demonstrated a sensitivity of 92.5% (95% CI, 84.6–96.5%) and a specificity of 99.6% (95% CI, 97.5–99.9%). Dauner et al. utilized turbidity for result analysis, while Teoh et al. employed fluorescence detection [13,14].

Our findings emphasize the need for further investigation and optimization of molecular diagnostic methods for DENV in specific populations. The variability in the efficacy of RT-LAMP protocols according to geographic and demographic contexts emphasizes the importance of validating these techniques in local cohorts before their widespread implementation in public health programs. Furthermore, the integration of complementary methods and a more comprehensive approach for detecting all DENV serotypes in populations with high genetic diversity and different levels of endemicity should be considered.

LAMP is a highly robust assay, allowing it to be performed even without extensive sample preparation in scenarios where false negatives due to low-copy-number samples are acceptable. It is estimated that individuals with acute dengue have varying viral loads, ranging from 10^3^ to 10^12^ plaque-forming units per milliliter [22]. These high viral titers can be detected by LAMP even without sample preparation in advance. We evaluated the TURN-RT-LAMP assay in crude samples (with no previous RNA extraction) and found that LAMP was sensitive to detect DENV without previous RNA extraction (Figure S1, Supplementary Materials).

Asymptomatic (nonfebrile) individuals with dengue exhibit viral loads similar to those of symptomatic individuals [24]. Moreover, the decay rate of viral RNA in asymptomatic individuals is slower. This emphasizes the potential of the LAMP test to be used as a screening tool in dengue-endemic areas, serving as a strategy for active dengue surveillance.

The discrepancies observed between the RT-LAMP and RT-qPCR results can be attributed to various factors, including differences in viral load among samples, the efficiency of RNA extraction, and the specificity of the primers for the different DENV serotypes. Using degenerate primers can help mitigate some of these challenges, enabling the detection of multiple serotypes in a single reaction, highlighting the need for further RT-LAMP optimization. The variability in RT-LAMP efficacy encourages further optimization of the protocol [22]. The results of our study are consistent with those of the reference test.

In our study, the detection limit of the TURN-RT-LAMP technique was approximately 800 viral copies for all DENV serotypes, using the primers reported by Teoh et al. [12]. In contrast, Zhou et al. reported varying detection limits for the four serotypes using the same primers: 74 copies for DENV-1, 252 copies for DENV-2, 78 copies for DENV-3, and 35 copies for DENV-4 [23]. They achieved this sensitivity by incorporating a high-fidelity DNA polymerase with 3′–5′ exonuclease activity, which reduced the error rate. Consequently, the Bst polymerase extension was utilized to establish a novel mismatch-tolerant RT-LAMP system [23]. Additionally, Hu et al. employed the RT-LAMP assay, detecting 100% of clinical strains and 98.9% of DENV-infected patient samples without any false positives [17]. The detection limit for DENV-1–4 was approximately 10 template copies, making this method 10 times more sensitive than RT-PCR or real-time PCR. Different target regions were used for each DENV serotype: NS2A for DENV-1, NS4B for DENV-2, NS4A for DENV-3, and the 3′-untranslated region of the NS protein for DENV-4 [17]. This protocol employed an isothermal temperature of 63 °C for 45 min, whereas our method used 65 °C for 30 min. Parida et al. used primers targeting the 3′ noncoding region (NCR) [21], achieving 100% sensitivity in detecting viral RNA in patient serum samples, with 93% specificity. Their RT-LAMP was conducted at 63 °C for 1 h using a Loopamp real-time turbidimeter. Lau et al. also targeted the NCR to detect DENV in real-time, developing a real-time RT-LAMP using the Loopamp real-time turbidimeter. Positive results were observed when turbidity reached 0.1 within 60 min at 650 nm. This method used an isothermal temperature of 65 °C with a 30 min incubation time for DENV-1, DENV-2, and DENV-3, and 45 min for DENV-4. This study was the first to incorporate HNB dye into the RT-LAMP assay for real-time DENV detection, demonstrating high sensitivity and specificity compared with ELISA and qRT-PCR, with a detection limit of 10 template copies [18]. Li et al. developed an RT-LAMP assay capable of simultaneously detecting DENV-1–4 serotypes, Japanese encephalitis virus, and West Nile virus. Using primers reported by Parida et al., they performed the assay at 63 °C for 15–30 min. The RT-LAMP assay demonstrated detection limits 100 times more sensitive than RT-PCR. Testing was conducted on sera from 168 patients and 279 pools of field-caught, blood-fed mosquitoes, showing complete concordance between RT-LAMP and RT-PCR results [19].

The development of portable sequencing methods together with isothermal amplification is a novel method reported in 2017 in Indonesian patient samples. The use of both methodologies allowed amplifying and sequencing the viral genome easier than the conventional techniques reported previously, with a high sensitivity and specificity. This would be further evidence that LAMP is a better, rapid, reliable, and sensitive method for genotyping [23].

Finally, our results are consistent with findings from other studies that emphasize the importance of assay characteristics such as robustness and ease of use in resource-limited settings. In such environments, methods such as RT-LAMP can be especially valuable. The ability of the TURN-RT-LAMP assay to be effectively conducted at an isothermal temperature and without the need for extensive sample preparation makes it a practical option for use in rural areas or during outbreak situations where access to well-equipped laboratories is limited. Our results demonstrate that the reaction reliably proceeds at 65 °C, making it suitable for use with a water bath or electricity-free heating devices. The assay’s colorimetric nature simplifies result interpretation, particularly in resource-limited settings where a UV light source may not be available. LAMP offers several advantages over traditional PCR, including reduced sensitivity to inhibitors such as ethanol, isopropanol, EDTA, and sodium acetate [25]. Additionally, the use of loop-specific primers can reduce assay times, providing faster results compared to PCR, and previous studies have reported higher sensitivity for LAMP [9]. While the multiplex LAMP technique is a promising tool for the rapid and simultaneous detection of multiple pathogens, several limitations must be addressed. These include its sensitivity to primer mismatches, which can result in false negatives, and the potential for false positives due to non-specific amplifications. Moreover, the design and optimization of primers in multiplex reactions present significant challenges, increasing the complexity of assay development. Minimizing contamination and standardizing reaction conditions are also essential to ensure reproducible results. Recognizing and addressing these limitations is critical to enhancing the robustness and clinical applicability of this technique. Furthermore, the assay is more convenient for diagnosing DENV because it targets the four serotypes in the same sample.

## 5. Conclusions

The TURN-RT-LAMP test was confirmed as a confident strategy for the molecular diagnosis of DENV in Colombia. It was much faster than PCR and demonstrated promising competitive results, which were confirmed by the PCR results.

Both the sensitivity and specificity of the TURN-RT-LAMP technique for the molecular diagnosis of DENV were 0.96. The positive predictive value was 0.91, and the negative predictive value was 0.98. This result indicates that the two sets of primers used to detect the 5′- and 3′-UTR regions of the four DENV serotypes were specific for amplifying the region of interest and diagnosing DENV in the samples.

## Figures and Tables

**Table 1:** Primer sequences of RT-qPCR.

Serotype	Primers	Sequences (5′-->3′)	Fluorophore	Target
DENV-1	DENV-1 F	CAAAAGGAAGTCGYGCAATA		NS5
	DENV-1 R	CTGAGTGAATTCTCTCTGCTRAAC		
	DENV-1 P	CATGTGGYTGGGAGCRCGC	FAM	
DENV-2	DENV-2 F	CAGGCTATGGCACYGTCACGAT		E
	DENV-2 R	CCATYTGCAGCARCACCATCTC		
	DENV-2 P	CTCYCCRAGAACGGGCCTCGACTTCAA	HEX	
DENV-3	DENV-3 F	GGACTRGACACACGCACCCA		prM
	DENV-3 R	CATGTCTCTACCTTCTCGACTTGYCT		
	DENV-3 P	ACCTGGATGTCGGCTGAAGGAGCTTG	TR	
DENV-4	DENV-4 F	TTGTCCTAATGATGCTRGTCG		prM
	DENV-4 P	TCCACCYGAGACTCCTTCCA		
	DENV-4 P	TYCCTACYCCTACGCATCGCATTCCG	Cy5	

**Table 2:** Sequences of the primers used in this study. Set 1 and Set 2 were previously reported. Primer concentration in the reaction, showing the initial and final concentrations in the LAMP reaction.

Primer LAMP Set	Oligos Name	Primer Sequence	10× Working Solution	1× Final Concentration in the LAMP Reaction
Set 1 [1]	FIP-DENV1/3	5′-GGTTATTCATCAGGATCTGCTCTCTTTTTTTCGAATCGGAAGCTTCGT-3′	16 μM	1.6 μM
	FIP-DENV2	5′-GGTTATTCATCAGAGATCTGCTCTCTTTATTCTTTGAGGGAGCTAAGC-3′		
	FIP-DENV4	5′-TTCATTTTTCCAGGATCTGCTCTCTTTTTTTCGAATCGGAAGCTTCGT-3′		
	B1P-DENV1/3	5′-AACGGAAAAAGACGGGTCAACCGTTTTTCTTTGTCAGCTGTTGCACAGT-3′	8 μM	0.8 μM
	B1P-DENV2	5′-AACGGAAAAAGGCGAGAAA TACGCTTTTCTTTGTCAGCTGTTGCACAGT-3′		
	B1P DENV4	5′-AACGAAAAAAGGTGGTTAGACCACTTTTCTTCACCAACCCTTGAGGGGT-3′		
	F3	5′-GTGGACCGACAAAGACAG-3′	2 μM	0.2 μM
	B3 DENV1/3	5′-GTGAGCAATCCTTTTGAG-3′		
	B3 DENV2	5′-TGCAGCA TTCCAAGTGAG-3′		
	B3 DENV4	5′-GAAAAAAGTCCGGTTGAG-3′		
	LoopB	5′-GCGAGAGAAACCGCGTGTC-3′	8 μM	0.8 μM
Set 2 [2]	F3/134	5′-CAAACCGTGCTGCCTGT-3′	2 μM	0.2 μM
	F3/2	5′-TGAGTAAACTATGCAGCCTGT-3′		
	B3/123	5′-ACCTGTTGA TTCAACAGCACC-3′		
	B3/4	5′-ACCTGTTGGATCAACAACACC· 3′		
	FIP/123	5′-AGGGGTCTCCTCT AACCRCTAGTCTTTCAAACCRTGGAAGCTGTACGC-3′	13 μM	1.3 μM
	FIP/4	5′AGGGGTCTCCTCTAACCRCTAGTCTTTTTTGCCACGGAAGCTGTACGC-3′		
	B1P/123	5′-ACAGCATATTGACGCTGGGARAGACGTTCTGTGCCTGGAA TGATGCTG-3′		
	B1P/4	5′-ACAGCATATTGACGCTGGGARAGACGCTCTGTGCCTGGATTGATGTTG-3′		
	BLP/1234	5′-CAGAGATCCTGCTGTCTC-3′	8 μM	0.8 μM

**Table 3:** Kappa index interpretation.

Kappa Values	Interpretation
<0	No agreement
0–0.19	Poor agreement
0.20–0.39	Fair agreement
0.40–0.59	Moderate agreement
0.60–0.79	Substantial agreement
0.80–1.00	Almost perfect agreement

**Table 4:** Copy number in relation to Cq RT-qPCR. Standard curves were generated using DENV-2, which was diluted from 10 ng/μL. The table illustrates the copy number and Cq for each dilution.

DENV-2	79 pb	Copy Number	Cq	desv Cq	Log Number Copy
Dilution Factor	ng/μL				
1	10	1,129,574	11	0.15	6
2	1	83,378	15	0.11	5
3	0.1	11,807	19	0.01	4
4	0.01	872	22	0.15	3
5	0.001	64	26	0.23	2
6	0.0001	9	29	0.19	1
7	0.00001	1	33	0.13	0
8	0.000001	0	35	1.29	−1

**Table 5:** Contingency table. This table shows 45 positive and 107 negative samples for both tests, 6 samples positive for TURN-RT-LAMP but negative for RT-qPCR, and 2 samples negative for TURN-RT-LAMP when the result of the reference is positive.

Table 2 × 2	Positive RT-qPCR	Negative RT-qPCR	Total
Positive TURN-RT-LAMP	45	6	51
Negative TURN-RT-LAMP	2	105	107
Total	47	111	158

**Table 6:** Sensitivity and specificity of the TURN-RT-LAMP test for DENV diagnosis. The sensitivity and specificity of the TURN-RT-LAMP test were 0.96 and 0.95, respectively. The concordance between both tests showed almost perfect agreement, with the value being 0.88.

Sensitivity	Specificity	Positive Predictive Value	Negative Predictive Value	Kappa Index	Po	Pe
0.96 (0.85, 0.99)	0.95 (0.89, 0.98)	0.88 (0.76, 0.96)	0.98 (0.93, 1.00)	0.88	0.949	0.571

**Table 7:** Sensitivity in relation to copy number. The table shows the range of copy number and the sensitivity of the TURN-RT-LAMP assay to each range. The concordance in samples with more than 1.3 × 10^2^ copies was perfect, and it showed almost perfect agreement in samples with 3 × 10^2^ copies to less than 1.3 × 10^2^ copies.

RT-qPCR			TURN-RT-LAMP			
**RT-qPCR**	**Copy Number**	**CT**	**Positives**	**Negatives**	**Total**	**Sensitivity**
	>1.3 × 10^2^	0–24	13	0	13	1
	1.3 × 10^2^–5	25–30	19	1	20	0.95
	<5	>30	19	5	24	0.79
**Total**			51	6	57	

## Data Availability

The original contributions presented in the study are included in the article/Supplementary Materials, further inquiries can be directed to the corresponding author.

## Supplementary Materials

The following supporting information can be downloaded at: https://www.mdpi.com/article/10.3390/diagnostics15050570/s1, Table S1: Results of DENV diagnostic tests; Figure S1: TURN-RT-LAMP performance with no RNA extraction.
