# Supplementary material for: Development and Validation of a Combined RT-LAMP Assay for the Rapid and Sensitive Detection of Dengue Virus in Clinical Samples from Colombia
Source: Diagnostics (Basel). 2025 Feb 27;15(5):570. doi: 10.3390/diagnostics15050570 (PMC11898505; doi:10.3390/diagnostics15050570)
Supplement: Supplementary file 1 [file diagnostics-15-00570-s001.zip › diagnostics-3216660-supplementary.pdf]

Supplementary Data

Supplementary Table S1. Results of DENV diagnostic tests. In this table is shown the information regarding to diagnostic tests of the samples and (Serology, RT-qPCR), copies number and Ct, DENV serotype and COMB-RT-LAMP results.

Code	Serology	RT-qPCR	CT	Copies number	Serotype	LAMP
DENV-CGN-001	IgM (+)	POSITIVE	21	1526	1	POSITIVE
DENV-CGN-002	IgM (+)	POSITIVE	22	654	2	POSITIVE
DENV-CGN-003	IgM (+)	POSITIVE	27	32	2	POSITIVE
DENV-CGN-004	IgM (+)	POSITIVE	30	4	2	POSITIVE
DENV-CGN-005	IgM (+)	POSITIVE	30	4	1	POSITIVE
DENV-CGN-006	IgM (+)	POSITIVE	18	11579	1	POSITIVO
DENV-CGN-007	IgM (+)	POSITIVE	23	339	1	POSITIVE
DENV-CGN-008	IgM (+)	POSITIVE	27	32	2	POSITIVE
DENV-CGN-009	IgM (+)	POSITIVE	23	352	1	POSITIVE
DENV-CGN-010	IgM (+)	POSITIVE	32	1	2	POSITIVE
DENV-CGN-011	IgM (+)	POSITIVE	30	6	1	POSITIVE
DENV-CGN-012	IgM (+)	POSITIVE	30	4	1	POSITIVE
DENV-CGN-013	IgM (+)	NEGATIVE	N.A	N.A	N.A	NEGATIVE
DENV-CGN-014	IgM (+)	POSITIVE	21	1808	1	POSITIVE
DENV-CGN-015	IgM (+)	POSITIVE	20	2928	2	POSITIVE
DENV-CGN-016	IgM (+)	POSITIVE	24	303	1	POSITIVE
DENV-CGN-017	IgM (+)	POSITIVE	28	23	1	POSITIVE
DENV-CGN-018	IgM (+)	POSITIVE	30	6	1	POSITIVE
DENV-CGN-019	IgM (+)	POSITIVE	30	5	1	NEGATIVE
DENV-CGN-020	IgM (+)	POSITIVE	31	2	1	NEGATIVE
DENV-CGN-021	IgM (+)	POSITIVE	24	192	1	POSITIVE
DENV-CGN-022	IgM (+)	NEGATIVE	N.A	NA	N.A	NEGATIVE
DENV-CGN-023	IgM (+)	NEGATIVE	N.A	N.A	N.A	NEGATIVE
DENV-CGN-024	IgM (+)	NEGATIVE	N.A	NA	N.A	NEGATIVE
DENV-CGN-025	IgM (+)	NEGATIVE	N.A	NA	N.A	NEGATIVE
DENV-CGN-026	IgM (+)	POSITIVE	34	0	1	NEGATIVE
DENV-CGN-027	IgM (+)	POSITIVE	28	16	1	POSITIVE
DENV-CGN-028	IgM (+)	POSITIVE	27	35	1	POSITIVE
DENV-CGN-029	IgM (+)	POSITIVE	25	136	2	POSITIVE
DENV-CGN-030	IgM (+)	POSITIVE	28	18	1	POSITIVE
DENV-CGN-031	IgM (+)	POSITIVE	27	25	1	POSITIVE
DENV-CGN-032	IgM (+)	POSITIVE	27	45	1	POSITIVE
DENV-CGN-033	NS1 (+)	POSITIVE	27	36	4	POSITIVE
DENV-CGN-034	NS1 (+)	NEGATIVE	N.A	NA	N.A	NEGATIVE
DENV-CGN-035	NS1 (+)	POSITIVE	30	5	4	POSITIVE
DENV-CGN-036	NS1 (+)	NEGATIVE	N.A	NA	N.A	NEGATIVE
DENV-CGN-037	NS1 (+)	NEGATIVE	N.A	NA	N.A	NEGATIVE
DENV-CGN-038	NS1 (+)	POSITIVE	32	2	3	NEGATIVE

DENV-CGN-039	NS1 (+)	POSITIVE	33	1	1	NEGATIVE
DENV-CGN-040	NS1 (-)	NEGATIVE	N.A	NA	N.A	NEGATIVE
DENV-CGN-041	NS1 (-)	NEGATIVE	N.A	NA	N.A	NEGATIVE
DENV-CGN-042	NS1 (-)	NEGATIVE	N.A	NA	N.A	NEGATIVE
DENV-CGN-043	NS1 (-)	NEGATIVE	N.A	NA	N.A	NEGATIVE
DENV-CGN-044	NS1 (-)	NEGATIVE	N.A	NA	N.A	NEGATIVE
DENV-CGN-045	NS1 (-)	NEGATIVE	N.A	NA	N.A	NEGATIVE
DENV-CGN-046	NS1 (-)	NEGATIVE	N.A	NA	N.A	NEGATIVE
DENV-CGN-047	NS1 (-)	NEGATIVE	N.A	NA	N.A	NEGATIVE
DENV-CGN-048	NS1 (-)	NEGATIVE	N.A	NA	N.A	NEGATIVE
DENV-CGN-049	NS1 (-)	NEGATIVE	N.A	NA	N.A	NEGATIVE
DENV-CGN-050	NS1 (-)	NEGATIVE	N.A	NA	N.A	NEGATIVE
DENV-CGN-051	NS1 (-)	NEGATIVE	N.A	NA	N.A	NEGATIVE
DENV-CGN-052	NS1 (-)	NEGATIVE	N.A	NA	N.A	NEGATIVE
DENV-CGN-053	IgM (+)	NEGATIVE	N.A	NA	N.A	NEGATIVE
DENV-CGN-054	IgM (+)	NEGATIVE	N.A	NA	N.A	NEGATIVE
DENV-CGN-055	IgM (+)	NEGATIVE	N.A	NA	N.A	NEGATIVE
DENV-CGN-056	IgM (+)	NEGATIVE	N.A	NA	N.A	NEGATIVE
DENV-CGN-057	IgM (+)	NEGATIVE	N.A	NA	N.A	NEGATIVE
DENV-CGN-058	IgM (+)	NEGATIVE	N.A	NA	N.A	NEGATIVE
DENV-CGN-059	IgM (+)	NEGATIVE	N.A	NA	N.A	NEGATIVE
DENV-CGN-060	IgM (+)	NEGATIVE	N.A	NA	N.A	NEGATIVE
DENV-CGN-061	IgM (+)	NEGATIVE	N.A	NA	N.A	NEGATIVE
DENV-CGN-062	IgM (+)	NEGATIVE	N.A	NA	N.A	NEGATIVE
DENV-CGN-063	IgM (+)	NEGATIVE	N.A	NA	N.A	NEGATIVE
DENV-CGN-064	IgM (+)	NEGATIVE	N.A	NA	N.A	NEGATIVE
DENV-CGN-065	IgM (+)	NEGATIVE	N.A	NA	N.A	NEGATIVE
DENV-CGN-066	IgM (-)	NEGATIVE	N.A	NA	N.A	NEGATIVE
DENV-CGN-067	IgM (-)	NEGATIVE	N.A	NA	N.A	NEGATIVE
DENV-CGN-068	IgM (-)	NEGATIVE	N.A	NA	N.A	NEGATIVE
DENV-CGN-069	IgM (-)	NEGATIVE	N.A	NA	N.A	NEGATIVE
DENV-CGN-070	IgM (-)	NEGATIVE	N.A	NA	N.A	NEGATIVE
DENV-CGN-071	IgM (-)	NEGATIVE	N.A	NA	N.A	NEGATIVE
DENV-CGN-072	IgM (-)	NEGATIVE	N.A	NA	N.A	NEGATIVE
DENV-CGN-073	IgM (-)	NEGATIVE	N.A	NA	N.A	NEGATIVE
DENV-CGN-074	NS1 (-)	NEGATIVE	N.A	NA	N.A	NEGATIVE
DENV-CGN-075	IgM (+)	NEGATIVE	N.A	NA	N.A	NEGATIVE
DENV-CGN-076	NS1 (+)	POSITIVE	24	309	4	POSITIVE
DENV-CGN-077	IgM (-)	NEGATIVE	N.A	N.A	N.A	NEGATIVE
DENV-CGN-078	IgM (-)	NEGATIVE	N.A	N.A	N.A	NEGATIVE
DENV-CGN-079	NS1 (+)	POSITIVE	28	21	4	POSITIVE
DENV-CGN-080	NS1 (-)	NEGATIVE	N.A	N.A	N.A	NEGATIVE
DENV-CGN-081	NS1 (-)	NEGATIVE	N.A	N.A	N.A	NEGATIVE
DENV-CGN-082	IgM (+)	NEGATIVE	N.A	N.A	N.A	NEGATIVE
DENV-CGN-083	NS1 (-)	NEGATIVE	N.A	N.A	N.A	NEGATIVE
DENV-CGN-084	NS1 (+)	NEGATIVE	N.A	N.A	N.A	NEGATIVE
DENV-CGN-085	NS1 (-)	NEGATIVE	N.A	N.A	N.A	NEGATIVE

DENV-CGN-086	IgM (+)	NEGATIVE	N.A	N.A	N.A	NEGATIVE
DENV-CGN-087	IgM (-)	NEGATIVE	N.A	N.A	N.A	NEGATIVE
DENV-CGN-088	NS1 (-)	NEGATIVE	N.A	N.A	N.A	POSITIVE
DENV-CGN-089	IgM (+)	NEGATIVE	N.A	N.A	N.A	NEGATIVE
DENV-CGN-090	IgM (-)	NEGATIVE	N.A	N.A	N.A	NEGATIVE
DENV-CGN-091	NS1 (-)	POSITIVE	33	1	3	POSITIVE
DENV-CGN-092	IgM (-)	POSITIVE	27	28	4	POSITIVE
DENV-CGN-093	NS1 (+)	POSITIVE	30	6	1	POSITIVE
DENV-CGN-094	IgM (-)	NEGATIVE	N.A	N.A	N.A	NEGATIVE
DENV-CGN-095	NS1 (-)	POSITIVE	32	1	2	POSITIVE
DENV-CGN-096	IgM (-)	NEGATIVE	N.A	N.A	N.A	NEGATIVE
DENV-CGN-097	NS1 (+)	POSITIVE	34	0	2	NEGATIVE
DENV-CGN-098	NS1 (-)	NEGATIVE	N.A	N.A	N.A	POSITIVE
DENV-CGN-099	IgM (-)	NEGATIVE	N.A	N.A	N.A	NEGATIVE
DENV-CGN-100	NS1 (-)	NEGATIVE	N.A	N.A	N.A	NEGATIVE
DENV-CGN-101	NS1 (-)	POSITIVE	32	1	2	POSITIVE
DENV-CGN-102	IgM (-)	NEGATIVE	N.A	N.A	N.A	NEGATIVE
DENV-CGN-103	IgM (-)	POSITIVE	31	2	2	POSITIVE
DENV-CGN-104	NS1 (-)	POSITIVE	32	1	1	POSITIVE
DENV-CGN-105	NS1 (-)	NEGATIVE	N.A	N.A	N.A	NEGATIVE
DENV-CGN-106	NS1(+)	NEGATIVE	N.A	N.A	N.A	NEGATIVE
DENV-CGN-107	NS1(+)	NEGATIVE	N.A	N.A	N.A	NEGATIVE
DENV-CGN-108	NS1(+)	NEGATIVE	N.A	N.A	N.A	NEGATIVE
DENV-CGN-109	NS1(+)	NEGATIVE	N.A	N.A	N.A	NEGATIVE
DENV-CGN-110	NS1(+)	POSITIVE	28.82	10	4	POSITIVE
DENV-CGN-111	NS1(+)	NEGATIVE	N.A	N.A	N.A	NEGATIVE
DENV-CGN-112	IgM(+)	NEGATIVE	N.A	N.A	N.A	NEGATIVE
DENV-CGN-113	IgM(+)	NEGATIVE	N.A	N.A	N.A	NEGATIVE
DENV-CGN-114	NS1(+)	NEGATIVE	N.A	N.A	N.A	NEGATIVE
DENV-CGN-115	IgM(+)	NEGATIVE	N.A	N.A	N.A	NEGATIVE
DENV-CGN-116	IgM(+)	NEGATIVE	N.A	N.A	N.A	NEGATIVE
DENV-CGN-117	NS1(+)	NEGATIVE	N.A	N.A	N.A	NEGATIVE
DENV-CGN-118	IgM(+)	NEGATIVE	N.A	N.A	N.A	NEGATIVE
DENV-CGN-119	IgM(+)	NEGATIVE	N.A	N.A	N.A	NEGATIVE
DENV-CGN-120	IgM(+)	NEGATIVE	N.A	N.A	N.A	NEGATIVE
DENV-CGN-121	NS1(+)	POSITIVE	30	4	4	POSITIVE
DENV-CGN-122	NS1(+)	POSITIVE	25	104	1	POSITIVE
DENV-CGN-123	NS1(+)	POSITIVE	31	2	4	POSITIVE
DENV-CGN-124	IgM(+)	NEGATIVE	N.A	N.A	N.A	NEGATIVE
DENV-CGN-125	NS1(+)	NEGATIVE	N.A	N.A	N.A	NEGATIVE
DENV-CGN-126	IgM(+)	NEGATIVE	N.A	N.A	N.A	NEGATIVE
DENV-CGN-127	NS1(+)	NEGATIVE	N.A	N.A	N.A	NEGATIVE
DENV-CGN-128	IgM(+)	NEGATIVE	N.A	N.A	N.A	NEGATIVE
DENV-CGN-129	NS1(+)	NEGATIVE	N.A	N.A	N.A	NEGATIVE
DENV-CGN-130	IgM(+)	NEGATIVE	N.A	N.A	N.A	NEGATIVE
DENV-CGN-131	NS1(+)	NEGATIVE	N.A	N.A	N.A	NEGATIVE
DENV-CGN-132	IgM(+)	NEGATIVE	N.A	N.A	N.A	NEGATIVE

DENV-CGN-133	IgM(+)	NEGATIVE	N.A	N.A	N.A	NEGATIVE
DENV-CGN-134	NS1(+)	NEGATIVE	N.A	N.A	N.A	NEGATIVE
DENV-CGN-135	NS1(+)	NEGATIVE	N.A	N.A	N.A	NEGATIVE
DENV-CGN-136	NS1(+)	NEGATIVE	N.A	N.A	N.A	NEGATIVE
DENV-CGN-137	IgM(+)	NEGATIVE	N.A	N.A	N.A	NEGATIVE
DENV-CGN-138	NS1(+)	POSITIVE	33	1	2	POSITIVE
DENV-CGN-139	NS1(+)	NEGATIVE	N.A	N.A	N.A	NEGATIVE
DENV-CGN-140	IgM(+)	NEGATIVE	N.A	N.A	N.A	NEGATIVE
DENV-CGN-141	IgM(+)	NEGATIVE	N.A	N.A	N.A	NEGATIVE
DENV-CGN-142	IgM(-)	NEGATIVE	N.A	N.A	N.A	NEGATIVE
DENV-CGN-143	NS1(+)	NEGATIVE	N.A	N.A	N.A	NEGATIVE
DENV-CGN-144	IgM(+)	NEGATIVE	N.A	N.A	N.A	NEGATIVE
DENV-CGN-145	NS1(+)	NEGATIVE	N.A	N.A	N.A	NEGATIVE
DENV-CGN-146	NS1(+)	NEGATIVE	N.A	N.A	N.A	NEGATIVE
DENV-CGN-147	IgM(+)	POSITIVE	33	1	2	POSITIVE
DENV-CGN-148	IgM(+)	NEGATIVE	N.A	N.A	N.A	NEGATIVE
DENV-CGN-149	IgM(+)	NEGATIVE	N.A	N.A	N.A	NEGATIVE
DENV-CGN-150	NS1(+)	POSITIVE	32	1	2	POSITIVE
DENV-CGN-151	NS1(+)	NEGATIVE	N.A	N.A	N.A	NEGATIVE
DENV-CGN-152	IgM(+)	POSITIVE	22	872	4	POSITIVE
DENV-CGN-153	NS1(+)	NEGATIVE	N.A	N.A	N.A	NEGATIVE
DENV-CGN-154	NS1(+)	POSITIVE	18	11807	4	POSITIVE
DENV-CGN-155	IgM(+)	POSITIVE	31	2	2	POSITIVE
DENV-CGN-156	IgM(+)	NEGATIVE	N.A	N.A	N.A	NEGATIVE
DENV-CGN-157	NS1(+)	NEGATIVE	N.A	N.A	N.A	NEGATIVE
DENV-CGN-158	IgM(+)	NEGATIVE	N.A	N.A	N.A	NEGATIVE

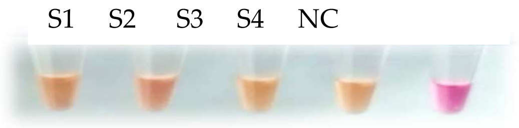

Supplementary Figure S1. TURN-RT-LAMP performance with no RNA extraction. Blood Samples from seropositive patients (S1-S4) were analyzed by the TURN-RT-LAMP test without previous RNA extraction. NC: Negative control.
